# Discharge Planning of Older Persons from Hospital: Comparison of Observed Practice to Recommended Best Practice

**DOI:** 10.3390/healthcare10020202

**Published:** 2022-01-20

**Authors:** Elise M. Gane, Veronika Schoeb, Petrea Cornwell, Cassandra Ranatunga Cooray, Brooke Cowie, Tracy A. Comans

**Affiliations:** 1 Centre for Allied Health Research, Metro North Hospital and Health Service, Brisbane 4006, Australia; e.gane@uq.edu.au (E.M.G.); cassandra.ranatunga@gmail.com (C.R.C.); 2 School of Health and Rehabilitation Sciences, The University of Queensland, Brisbane 4072, Australia; 3 Centre for Functioning and Health Research, Metro South Hospital and Health Service, Brisbane 4102, Australia; 4 Physiotherapy Department, Princess Alexandra Hospital, Brisbane 4102, Australia; 5 Department of Rehabilitation Sciences, The Hong Kong Polytechnic University, Hung Hom, Hong Kong; veronika.schoeb@hesav.ch; 6 School of Health Sciences (HESAV), University of Applied Sciences and Arts, Western Switzerland (HES-SO), 1011 Lausanne, Switzerland; 7 Menzies Health Institute Queensland, Griffith University, Brisbane 4222, Australia; p.cornwell@griffith.edu.au; 8 Caboolture Hospital, Metro North Hospital and Health Service, Caboolture 4510, Australia; brooke.cowie@health.qld.gov.au; 9 Centre for Health Services Research, The University of Queensland, Brisbane 4102, Australia

**Keywords:** discharge planning, communication, qualitative research, geriatric assessment, older adults, hospital organization

## Abstract

Older people are particularly vulnerable to hospital re-presentation following discharge. Ideal discharge planning processes facilitate the transition from hospital to home and prevent subsequent re-presentations to hospital. The objective of this study was to examine discharge planning processes in two Australian hospitals, compare them between sites and to best-practice recommendations. An ethnographic observational study of discharge planning processes was conducted at two general medical inpatient wards at a large tertiary hospital and a smaller regional hospital in Brisbane, Australia. Participants were patients and ward staff involved in discharge planning during a hospital admission. A literature review was conducted to elicit best-practice recommendations for discharge planning. Data for this study (duration: 112 h) were collected directly using field notes by a research assistant embedded in the ward. A directed qualitative content analysis approach was used for data analysis. Results were compared to best-practice recommendations. Findings indicate that both hospitals implemented various best-practice interventions to enhance communication, collaboration, coordination and patient/family engagement for optimal discharge planning. Strategies used were context specific and effective to varying degrees. Clear responsibilities and goals within the multidisciplinary team helped to create cohesive, well-functioning teams. More work is needed to engage patients and families in discharge planning, and to encourage health professionals to consider patients and family as active team members in the discharge planning process.

## 1. Introduction

There is increasing pressure on health systems as a result of the growing global trend for an ageing population. This trend translates into more aged persons being admitted to hospital and requiring medical treatment [1]. The daily mismatch between demand (admissions) and capacity (available beds)—although normally temporary—puts additional pressure on health care facilities [2]. With an older patient population comes a specific set of care needs, including the need for interaction with residential aged care facilities and carers who have responsibility for the patient’s welfare once discharged from hospital. 

Older people are particularly vulnerable to hospital re-presentation, either with complications of medical management, or with exacerbations of comorbid disease. Evidence from the United States suggests that the majority (90%) of hospital readmissions are unplanned [3]. Unplanned readmissions within 30 days of hospital discharge may reflect deficiencies in the planning of the initial discharge [4]. Discharge planning is the process of developing an individually tailored plan within a hospital setting to facilitate the return of individuals to their homes in the community following an inpatient admission [5]. Interventions that address more than one component of the discharge planning process are effective in reducing readmissions [4], and comprehensive discharge planning with older patients admitted to hospital can result in small reductions (<1 day) in hospital length of stay and unscheduled readmissions (~3 fewer readmissions per 100 patients) [5]. 

Many different models of discharge planning exist that include a variety of different health professionals, in hospital and the community, as well as the patient and their family or carers. Globally, there is no consensus on the best model of discharge planning. However, there are common key points for effective discharge planning procedures, such as promoting a whole-system approach with a multidisciplinary team (MDT) working towards a common goal in a patient-centered manner [6]. For successful discharge planning to occur, patients’ needs should be identified at admission, and collaboration with post-discharge services should occur to ensure a smooth transition [6,7]. 

Operations research is the study of resource allocation problems using mathematical models and has been applied mainly in health in emergency department and outpatient settings [8,9]. In application to discharge planning for older people, one study found efficiencies could be realized in reducing discharge time by better coordination of staff [10] and another study on post hip fracture modelled future resource needs [11]. While operations research is useful for identifying process improvement, models of best practice may or may not be achievable “on the ground” and therefore, it is worthwhile to compare wards and service recommendations to identify areas where improvement is needed. Furthermore, comparison between facilities in the same health service district, where executive oversight could be said to be consistent, could identify circumstances unique to the individual facility that may influence policy execution.

Therefore, the aims of this study were to: (1) observe the discharge planning processes employed with older patients at two hospitals within the same health service district, (2) compare their practice to evidence-based recommendations for discharge planning, and (3) compare their practice with each other.

## 2. Materials and Methods

### 2.1. Study Design and Setting

This study was part of a mixed-methods research project evaluating discharge planning processes in a major health service in Brisbane, Australia. This health service serves approximately one million residents with five hospitals and various community health centers. The present report describes an ethnographic study of two acute medical inpatient wards. Each ward consisted of 30 beds and would manage on average around 100 patients per week. One setting was a large tertiary hospital and the other a smaller regional hospital; chosen because they were already using identifiable and specific discharge procedures. Commonly managed diagnoses on the two wards included but were not limited to: congestive heart failure, acute exacerbation of chronic obstructive pulmonary disease, and cellulitis. Analyzing discharge planning in two hospitals within the same jurisdiction but with different size and organizational structures might allow for the identification of key aspects of their culture within each facility. Ethnography is particularly well suited to describing the culture of an institution and understanding health care teamwork [12]. It seeks to explain both explicit aspects of a culture (what all members are aware of and take for granted), and tacit elements (outside of awareness), often manifested in nods, silences, or other communicative subtleties [13].

### 2.2. Participants

Members of the MDT in the selected hospitals took part in this study. Prior to data collection, senior research team members met with the nurse unit manager and other key staff members on each ward to clarify scope and mutual expectations. Ethical approval to conduct this study was given by two Institutional Review Boards, one hospital based (reference number HREC/16/QPCH/34), and one university based (reference number 2016/317). Ten older patients (>65 year), likely to stay more than five days in hospital, and able to give informed consent in each ward were individually recruited to take part in this study. Each patient provided written informed consent. Patients were purposely selected to represent the range of conditions usually seen in the unit with consultation with the team leader of the ward. Observation of staff at the hospital was approved under a waiver of consent (i.e., individual staff members did not provide informed consent). MDT members (hereafter referred to as clinicians) on the wards included medical officers (consultants, registrars, residents, interns, medical students); nurses (discharge coordinators, shift team leaders, clinical nurses, other specialist nurse roles); allied health professionals (physiotherapists, occupational therapists, speech therapists, social workers, dieticians, psychologists, pharmacists, allied health assistants); and administration staff. This study was conducted in accordance with the principles of the Declaration of Helsinki.

### 2.3. Data Collection

A research assistant with a background in public health and qualitative research, and no record of employment within the health service, was embedded in each ward and took field notes from direct observations of formal and informal discharge-related discussions between clinicians and between clinicians and patients of the patients recruited to this study as well as broader observations from team meetings. Field observations took place on each ward for six to eight working days (i.e., Monday to Friday), for a combined total of approximately 112 h, producing a combined total of 42 pages of field notes, providing insights into interactions and capturing context and processes [14]. This volume of data collection is consistent with previous examples of ethnographic research in health care settings [15].

### 2.4. Data Analysis

Field notes were transcribed from pen/paper into electronic form and qualitative content analysis; specifically, a directed approach was used by author EG to analyze the data. This approach was chosen in order to “validate or extend conceptually (the) theoretical framework”, i.e., discharge planning [16]. The predetermined codes used for this directed content analysis were derived from Carroll and Dowling’s essential elements for discharge planning [6] (see Supplementary Material S1 for details):

1. Communication: health professional to patient, family/carer, or community service;
2. Collaboration: between health professionals;
3. Coordination and Education: a designated health professional coordinates the discharge process and provides education to all parties to facilitate the process;
4. Patient participation: in their own discharge planning process; consideration of patient satisfaction from the process.

The coded data were compiled to make a comparison, first, between the two hospitals, and secondly, between the hospitals and the gold standard framework. Results and discussion of those results are presented together, followed by implications for practice and research, as per previously published guidelines for qualitative manuscripts [17].

Scientific rigor is essential for good qualitative research. According to Petty, Thomson [18], four criteria need to be applied: (a) confirmability (e.g., member checking, audit trail); (b) dependability (e.g., triangulation, reflexivity); (c) credibility (e.g., prolonged engagement, peer debriefing); and (d) transferability (e.g., purposeful sampling, thick description). During data collection, prolonged engagement in the field ensured credibility, while during data analysis peer debriefing and triangulation enhanced the understanding of variations within the two sites (dependability). Regular review and reflection on how author EG was coding the transcripts by author VS was also employed during data analysis to achieve confirmability. In order to allow transferability of the findings to other contexts, description of the study context and the observed situation (including citations) was provided, as suggested by Petty et al. [18].

## 3. Results

The presentation of results is in four sections, which are the four essential elements of discharge planning (used here as the evidence-based guidelines) from Carroll and Dowling (2007). Each essential element has results presented firstly from the tertiary hospital, then the regional hospital, followed by an interpretation of the data by means of a comparison between the hospitals.

### 3.1. Communication with Patients, Family/Carers, and Community Services

The Discharge Care Coordinator (DCC) at the **tertiary hospital** was a nurse with specialized skills in the coordination of both community services and members of the MDT within the hospital. The DCC would meet with all patients and make contact with family/carers in planning for discharge. Occasionally patients were asked the same questions by multiple clinicians conducting their own standalone assessments. To address this problem, and to facilitate communication more broadly, a meeting between multiple clinicians, the patient and their family/carers could be arranged, referred to as a *“family meeting”* Despite this, evidence of actively engaging the patient and family/carer in discharge planning was minimal.

Two of the services that tertiary hospital clinicians regularly engaged with were slow-stream rehabilitation facilities. Clinicians admitted being confused as to why some patients were transferred to these facilities on weekends. Senior management staff were perceived by clinicians to focus on *“bed pressures”* and to misunderstand the complex nature of some patient cases. The following text is an observation from the research assistant at the tertiary hospital (field notes from tertiary hospital, page 11, lines 2–10):

In morning MDT meeting–noted a number of discharges/changes over weekend–people who were not quite ready for it, were sent to [the slow-stream rehabilitation facility], including one patient who [the social worker] referred to as an inappropriate person to send. Confusion between [facility 1] and [facility 2] wanting/taking/being ready to take the people. After meeting I asked one social worker–who told me it is likely because of bed pressures. Executive would have sent people with the longer length of stay, but don’t realize that there is a reason for longer length of stay–often more complicated cases. Really not ready to go. Social worker: *“Really inappropriate people taken to [facility] on Saturday…the other thing is, if he was going on Saturday, why was he not sent on Friday?”*

There was a difference in referral procedures across community services whom the staff at the tertiary hospital regularly interacted with (e.g., telephone, time-consuming online referrals). A large portion of the DCC’s time was spent organizing relevant referrals in preparation for discharge. Referral plans were shared with the wider MDT via the medical chart and morning meetings. In some cases, a number of different assessments by various organizations or professionals were required prior to discharge. Length of stay could be increased whilst necessary assessments took place. Community primary care practitioners received written discharge summaries prepared by the relevant medical team.

On several occasions at the **regional hospital**, clinicians were observed to champion the wishes of patients and their families/carers after having discussed these with them. Family meetings could be requested by the medical team or DCC, and were arranged by the social worker. Family meetings were seen as a method of addressing situations in which patients or families were resisting or delaying discharge. However, clinicians did perceive family meetings as time intensive. Outside of a family meeting, communication between clinicians and patients could be conflicting. There were expectations of certain professions to talk with certain parties: doctors spoke with patients; DCCs and nursing team leaders spoke with family members and community services.

Clinicians at the regional hospital who were familiar with other clinicians employed by community organizations found it easier to communicate directly with each other instead of via the service’s national head office, which was often located interstate and could delay the process. On a number of occasions, medical officers were observed to remark about the returning nature of some patients. One doctor was observed to say: *“I have a feeling we’ll see her back for nursing home placement.”* (field notes from regional hospital, page 17, line 11) Community services were discussed as a potential intervention to address readmissions. Another doctor stated: *“Next patient has para influenza, lives with grandson, significantly deconditioned. Probably needs services because she keeps coming back.”* (field notes from regional hospital, page 22, line 2).

Residential aged care facilities only admitted certain patients when a registered nurse was on site, as they were the only available staff authorized to administer analgesia such as morphine. This led to delays in discharges from the regional hospital or return of patients from facilities during the observation period. On the other hand, clinicians did comment when services were working well and discharges were executed effectively as a result.

#### Comparison between Hospitals

Both hospitals used family meetings to facilitate communication between staff, patients, and families/carers, and written discharge summaries as one-way information transfers to primary care physicians. Beyond these strategies, however, active engagement of patients, families/carers, or primary care physicians was minimal. The role of the DCC at the regional hospital was emphasized as the primary contact point for families/carers to discuss patient discharge, and at both hospitals as the primary liaison with community services. Familiarity between hospital and community services staff enhanced communication.

### 3.2. Collaboration between Health Professionals

At the tertiary hospital, morning board rounds were held daily (on weekdays) and were attended by all members of the MDT. This meeting was a collaborative meeting usually chaired by the social worker. A representative from each medical team provided an update followed by allied health clinicians providing their viewpoints on patient status or raising questions and commenting on proposed discharge dates. *“Tension between some staff and procedures”* was observed during this meeting, especially when medical staff either pushed for *“short and sweet”* discussions, or when they did not know a patient case well enough to provide answers to questions from other clinicians.

A key communication tool—an electronic patient journey board [19]—facilitated the sharing of written information pertinent to patient care and discharge between MDT members at the tertiary hospital. It was prominently displayed on a screen in the nurses’ station and was the central focus point of the daily ward meeting. Patient information was updated by the meeting chair in real time throughout the MDT discussion. This information was available immediately and easily accessed anywhere in the hospital. Many staff were observed consulting and updating the board frequently each day.

Beyond the formally appointed times for discharge planning discussions at the tertiary hospital, informal discussions between clinicians also occurred. In designated staff areas for nurses as well as other staff rooms, conversations arose from chance meetings between clinicians. In addition, they regularly engaged in telephone calls with each other to communicate assessment findings or plan preparatory meetings leading up to family meetings for particularly complex discharge cases. The following text is an observation of an interaction made by the research assistant at the tertiary hospital (field notes from tertiary hospital, page 14, lines 5–8):

When preparing for family meetings (complex cases) the ward team and med team have a pre-meeting meeting to clarify

Physio: *“I haven’t caught up with her husband yet so…”*

Occupational therapist: *“I did”* and so occupational therapist was able to describe husband’s wishes for her to return etc.

A variety of considerations needed to be accounted for during discharge planning by a number of different clinicians at the tertiary hospital. The necessary collaboration was challenged if medical teams presented to the meeting late, or not at all. For example, the research assistant observed at the tertiary hospital: *Board rounds for ward: Today all allied health staff were there, but had to call just about every medical team to remind them to come up* (field notes from tertiary hospital, page 6, lines 27–28). If medical teams did not attend at all, the allied health staff conducted the review of patients themselves: *allied health staff reviewed [medical team’s patients] by themselves without medical input as by 10 am still no one had arrived* (field notes from tertiary hospital, page 7, lines 1–2).

Within the medical profession at the tertiary hospital, the involvement of more than one medical team might delay discharge. As an alternative to remaining an inpatient for certain investigations, tests could sometimes be delayed and conducted as an outpatient. Doctors were observed to regularly acknowledge the need for nursing and allied health input into the discharge planning of patients, although differences of opinion on patient status did occur.

Nursing staff and allied health clinicians made up the majority of the MDT at the tertiary hospital. Senior experienced nurses were present in roles including DCC and nursing team leader. allied health clinicians engaged in discharge planning collaboratively, including conducting joint assessments. Social workers, in particular, had valuable information that could indicate likely delays to discharge in individual cases. The pharmacist also had a critical role in the dispensing of medications for discharge, a process which could delay the patient leaving the hospital on the day of discharge.

The **regional hospital** also employed a formal discharge planning meeting: daily (weekdays) Rapid Rounds meetings were attended by medical teams (who provided a meeting chair), allied health, and DCC, held in a room with tiered seating. Registrars or consultants presented patient information, including expected dates of discharge, and led a brief discussion of each case to meeting attendees. As patients were discussed in order according to their allocated medical team, other staff were required to quickly shuffle through printed patient ward lists to update their notes as the meeting went on. The Rapid Rounds meetings were a significant source of frustration for nursing and allied health staff because: (i) medical teams were often late or did not attend at all; and (ii) expected dates of discharge were rarely updated as a result of this meeting. A senior allied health clinician at the regional hospital was observed to say: *“The purpose of Rapid Rounds is to establish EDDs [estimated discharge date], not to discuss patients but at the moment neither EDDs, nor much discussion really happens effectively.”* (field notes from regional hospital, page 37, lines 14–15).

In contrast, when medical officers did meet staff expectations in chairing this meeting, it was appreciated. The research assistant observed at the regional hospital: *One younger Registrar actually does a great job of giving EDDs for every patient, and [Doctor] also focusses [their] comments quite specifically on discharge planning* (field notes from regional hospital, page 14, lines 4–10). Another doctor was observed emphasizing the need to set an estimated date of discharge for every patient, and acknowledging the expectation that every patient required an estimated date of discharge: *The [medical specialty] registrar is very good at giving EDDs, for example, [they] said:* *“Possible discharge today, but for the purpose of this thing we’ll say tomorrow.”* (field notes from regional hospital, page 20, lines 4–5).

The main tool for information sharing at the regional hospital was the patient medical chart. However, clinicians were observed to be frustrated when time was lost looking for charts when needed. The research assistant recorded the following observation: *Team leader (slightly frustrated in relation to access to and chart availability) said* *“This is one of our limitations.” The [staff member] I was with (at the nurses’ station) was looking at / or writing in the chart that the team leader needed.* (field notes from regional hospital, page 5, lines 19–21). There was an electronic patient journey board positioned behind the nurses’ reception desk, yet it was rarely accessed by staff. The less than ideal use of both of these communication tools might have hampered communication between clinicians. For example, one doctor was observed to recommend: *“I think physio review would be helpful as well.”* The physiotherapist replied immediately: *“I’ve seen him”* (field notes from regional hospital, page 36, lines 24–25).

Informal meetings in staff rooms or corridors did occur at the regional hospital. Different allied health clinicians had different workload allocations, ranging from a single ward to the whole hospital, resulting in some clinicians being difficult to locate when discussions were needed. One-on-one meetings to discuss specific patient cases were still organized as a result of the formal meetings.

Different allied health clinicians have different discharge planning roles within the MDT at the regional hospital, and sometimes these roles overlap. For example, the responsibilities of the DCC (involved in discharge to residential aged care) and social worker (liaison to Disability Services Queensland) were often confused. Furthermore, the DCC’s assessment of the patient also occasionally overlapped with the occupational therapy assessment (e.g., cognitive evaluation). There was a significant amount of confusion observed between the nursing team leader and the staff in the Transit Lounge (pre- and post-admission waiting area) regarding who was responsible for coordinating transport from hospital to home.

Allied health professionals were frequently observed to work collaboratively with each other at the regional hospital, whereas medical staff were observed to partake in brief discussions with clinicians from other professions. This brevity in communication led to some allied health staff feeling their views were being ignored. The consequence of this non-consideration of the views of allied health staff was that patients were discharged from hospital when medically stable, but not physically or cognitively safe to return home.

#### Comparison between Hospitals

There were considerable differences observed between the MDT discharge planning processes held at the two hospitals. Electronic patient journey boards were used frequently at the tertiary hospital while their use was limited at the regional hospital, resulting in a reliance on medical charts. At the tertiary hospital, morning board rounds were ward based, collaborative, and focused on discharge planning; whereas at the regional hospital, Rapid Rounds meetings were hospital-wide information-sharing that was monocratic in nature and ineffective at establishing estimated dates of discharge. One of the benefits of having a ward-based meeting was that fewer patients were discussed, and fewer staff were present. Those present were relevant to the care of patients on that specific ward. There were inefficiencies at the Rapid Rounds meetings with the need to shuffle through paper lists of patients, as well as the allied health assistant (who was controlling the projection of patient data onto the lecture room screen) having difficulty in keeping up with the discussion. This ineffective meeting method could have resulted from the use of a medical model, instead of a wider multidisciplinary model, as was the case at the tertiary hospital.

### 3.3. Coordination and Education via a Dedicated Coordinating Health Professional

Within the tertiary hospital, the DCC had responsibility for many of the referrals to community services (e.g., domiciliary nursing, household cleaning, meals preparation). Some community referrals required collaboration between the DCC and other members of the MDT. If the DCC was absent, there were written resources available for other clinicians to use. Similarly, there was a large collection of resources available for sharing between DCCs to assist with handing over patient cases to new staff members. In addition to the DCC, two other staff members had roles with intersecting responsibilities: (i) the Nurse Navigator, focused on the management of long-stay patients; and (ii) the Geriatric Liaison professional, whose specific role description was not clear during data collection on the ward.

As well as coordinating referrals to community services, DCCs at the tertiary hospital played a pivotal role in educating patients and their family/carers about discharge plans. The DCC bore the responsibility for contacting family/carers to explain what community services had been organized. The DCC was occasionally the very first hospital staff member to consult with family. Engaging families in the discharge planning process might involve family members actively looking for a suitable place at a residential aged care facility for a patient. The DCC was responsible for liaising between community services, patients, families and carers, and the hospital-based MDT to make sure delays to discharge could be avoided.

The DCC at the **regional hospital** coordinated community referrals and placed great emphasis on having a positive working relationship with staff in community services as a way of facilitating information sharing. When services had been organized to be in place prior to hospital discharge, confirmation was then given to the wider MDT by the DCC. To assist the discharge planning process from time of admission, DCCs would complete a “complex risk assessment” upon admission to the Emergency Department or immediately after admission to the inpatient ward. The following was observed by the research assistant at the regional hospital (field notes from tertiary hospital, page 9, lines 7–11):

Usually this assessment is done in [Emergency Department], DCCs up on the wards, will do it if it’s not done when the patients come up to this ward. This is a fairly comprehensive document covering the patient’s situation and includes a combination of medical and social needs. This is an example of how some processes in the hospital do involve some level of discharge planning from the start. The document has a series of questions and contains excellent and clear instruction for the DCC filling it out, on who to contact (allied health) and what to do-referrals, calls etc. at each step.

The DCC was seen as having a variety of responsibilities to different stakeholders, in comparison with other clinicians who may have had singular responsibilities (e.g., being aware of a patient’s social history). DCCs were the contact person for family members, and providers of information to carers regarding availability of respite services or financial support where appropriate. Despite the service provided by the DCCs being seen as valuable, some staff felt it was unfortunate that their services were not available full time or that the rostering of individual DCCs to specific wards was not consistent. One of the administrative officers was observed saying *“Can we just get one DCC?”*, referencing the pattern of DDCs rotating between wards every few days. The Team leader responded: *“I dunno, but they are not even here for a full week, it’s just a few days.”* (field notes from tertiary hospital, page 37, lines 25–27).

#### Comparison between Hospitals

DCCs were used as coordinators of discharge planning, educators of patients and their families/carers, and sources of information for other MDT members at both hospitals. In addition to the DCC, the regional hospital employed specific documentation to assist with discharge planning from time of admission. It was useful in both tertiary and regional settings to have the role of DCC within the MDT to perform these coordination and education tasks. In particular, it was useful for patients and families to have a clearly identified contact person to whom questions could be addressed. The DCC was more freely available than medical staff for discussions with patients and families, and better able to address concerns about the involvement of community services and residential aged care. In addition to having the role of DCC present within the MDT, other clinicians also found it useful to have consistency in the staff allocated to those roles and to particular wards.

### 3.4. Patient Participation

Observations made at both hospitals were limited to how clinicians discussed patient choices and behaviors, and did not include observations of family meetings or other interactions between clinicians and patients. It is from this information that these results are drawn.

The wishes and behaviors of patients at the **tertiary hospital** were often described by clinicians during discussions with their peers when planning discharge destinations (to home, the community or to inpatient rehabilitation). A patient’s preference to return to their own home was frequently discussed, as was consent from family members for community services to be organized. The social worker in particular played a key role among the allied health clinicians as a patient advocate and as the clinician who identifies when discharge might be delayed by complex social situations. This is evidenced by the following interaction observed at the tertiary hospital between a physiotherapist and a social worker, talking about a patient who the physiotherapist thought was a candidate for inpatient rehabilitation (field notes from tertiary hospital, page 10, lines 4–9):

Social worker: *“Well you know we disagree on that. He would prefer to go home. Though they’ve said he’s going to nursing [home].”*

Physiotherapist: *“I just want to know which way to push.”*

Social worker: *“Quality of life.”*

Physiotherapist: *“For whom?”*

Discussions of perspectives of various family members were observed between clinicians at the tertiary hospital. Spouses and carers were used as a reference point for what constituted patient baseline functioning. Family members were involved in trialing patients living at home if they were to take on long-term carer responsibilities. However, if services were unavailable to start when discharge had been expected, family members were also considered by staff as potential interim carers in these situations. DCCs provided extra written information regarding services on discharge to family members upon request, as captured in this observation (field notes from tertiary hospital, page 4, lines 1–3):

11.30 a.m. [The DCC] wrote notes in chart about discharge plans to let [other clinicians] know. Also wrote up a word document to let patient know what going on (not common practice, but this [patient] and daughter were quite demanding and so this should help make things clear).

Conversations were observed at the **regional hospital** between health professionals during which patients were described as wanting to be discharged to home when they were expected to fail, or refusing to be discharged when they were medically stable. Patients considered to be refusing nursing home placement needed to be discharged back to their homes, where they were expected to be unable to manage; at which point they would re-present to the hospital and might then be accepting of nursing home placement. One doctor said of a patient who wanted to discharge despite needing increasing home supports: *“Unfortunately he’ll be back, then maybe we can convince him about nursing home. He’s stubborn at home.”* (field notes from regional hospital, page 25, lines 27–28) Cases of patient self-discharge as well as patient refusal to be discharged were also observed. Sometimes family meetings were arranged, or specific team members spoke to the patient’s spouse in order to reach an agreement about when discharge would occur.

#### Comparison between Hospitals

Health professionals at both hospitals were observed to report the feelings and wishes of patients and/or their families/carers, indicating that they had engaged them in discussions on issues such as readiness to discharge and preferred discharge destination. Patient preference appeared to be considered a positive addition to discharge planning at the tertiary hospital. In contrast, patients were placed in a more passive position at the regional hospital, with their preference for discharging to the home being seen as something to be “proved wrong” in order for patients to accept nursing home placement.

Family members appeared to be involved in the discharge planning process in a different way between hospitals. Families of patients at the tertiary hospital were engaged in more face-to-face time with clinicians, whereas families of patients at the regional hospital were more likely to receive regular telephone updates from the DCC. Family meetings were a useful tool for multiple staff to engage patients and family members in the decision-making process. However, there is a cost to clinicians in time spent preparing for and attending them.

## 4. Discussion

### 4.1. Implications for Clinical Practice

Four key opportunities for improvement in clinical practice arose from the findings of this study: (i) making better use of electronic journey boards; (ii) facilitating casual conversations between staff on wards; (iii) utilizing a DCC; and (iv) championing patient and family engagement.

#### 4.1.1. Electronic Journey Boards

To promote a more interactive and collaborative approach to communication, adequate communication tools need to be present, as do sufficient institutional leadership and team processes. In contrast to medical charts which do not seem to favor collaboration, an interactive electronic journey board might be more useful as information (e.g., expected date of discharge) can be continuously updated and widely accessed. Clinical dashboards were introduced into health care in the last decade [20]. Dashboards, whether in cars, cockpits or hospitals, represent data visually that can be shared among professionals and includes real-time data of ward activities [21]. Furthermore, a standardized presentation of data with electronic forms may improve information sharing and, by extension, service delivery for high risk patients [22]. Digitalization in health care can bring solutions in favor of effective team communication and collaboration.

#### 4.1.2. Casual Conversations between Staff on the Ward

In the structured and hierarchical environment of a hospital, staff at the lower end of that hierarchy may feel reduced levels of psychological safety in formal multidisciplinary meetings, defined as “the degree to which people view the environment as conducive to interpersonally risky behaviors like speaking up.” [23]. This withholding of information is a barrier to effective patient care, adult learning and team-based improvement [24,25]. Promoting casual conversations between team members in spaces such as the nurses’ station, common staff break room, or even the corridor may offer a solution (in part or whole) to overcoming the lack of confidence some staff may feel in speaking up in a formal meeting setting where members of the upper hierarchy are present.

#### 4.1.3. The Discharge Care Coordinator

Utilizing the skills and knowledge of a DCC can assist both the MDT, as well as the patient and their family, to achieve a safe and effective discharge from hospital. Formalization of the role of DCC within a hospital or health service may require some reorganization of the workforce and workplace culture [26]. The DCC could oversee the use of a standardized structure for discharge planning of older patients, which is often used as an indicator of quality in health care [27,28]. They are also well placed to practice “leader inclusiveness” within the MDT, to encompass all team members in decision making and valuing diversity of opinion [29]. Finally, there is evidence to suggest that discharge coordination can engage older patients and their families in discharge planning, resulting in lower rates of rehospitalization [30].

#### 4.1.4. Patient and Family Engagement

There are steps that MDTs can take to promote inclusivity of older patients and their families in discharge planning, and to set up a culture in which the perspectives of older patients and their families are respected and emphasized as an important component of care giving by all members of the MDT. Patient and family/carer feedback can be integrated into hospital governance using a patient and family advisory council, resulting in use of collaborative models of care and improvements in quality of patient care and patient satisfaction [31]. At the bedside, patients and their caregivers want information about the treatment the patient is receiving [32]. Written information appropriate for older patients about common medical conditions or interventions could be used as a communication tool between health professionals and patients/family members. In addition, family members who are taking on caring roles want information about systems that can support them emotionally as well as practically [33]. The different aspects of being a carer should be discussed in family meetings to gauge the family’s understanding and preparedness for the role of caregiving. In addition, health professionals should avoid making assumptions about the willingness of family members to become carers before speaking to those concerned. Lastly, communication should address older people’s care needs, but also the changing nature of those care needs, which might vary with disease progression and patient goals [34]. Goal setting should not be overlooked as a method of engaging patients and their families in health care [35,36], and as a communication tool to engage the wider MDT in effective discharge planning.

### 4.2. Implications for Research

This study provided insights into processes of discharge planning, rather than focusing on the discharge outcome. It is argued that ethnographic studies can help describe the culture of an institution and be used for understanding health care teamwork [12]. Being in the field provides an opportunity to learn about the institution, formal and informal division of labor in the setting, and health professionals’ interactional patterns [37]. Using this observational approach, a social phenomenon can be explored and the meaning that participants give to a certain action can be identified as they go about their everyday lives [38].

As illustrated in this study, there is a gap between the evidence base and clinical practice [39]. One potential solution is to be methodical in the integration of new evidence-based initiatives into clinical care using the principles of implementation science. Implementation science is “the scientific study of methods to promote the systematic uptake of research findings and other evidence-based practices into routine practice, and, hence, to improve the quality and effectiveness of health services.” [40]. There are previously published examples of implementation science in the field of discharge planning of older persons, for example, the integration of a ‘nurse discharge navigator’ specifically for people with heart failure or sepsis [41]. In a scoping review that aimed to describe implementation issues in integrated care systems for older or frail clinical populations, authors reported four barriers to implementation: macro-level factors external to the health service, miso-level factors of system organization (funding models, organizational leadership), miso-level factors of intervention organization (intervention complexity, resources, and credibility), and micro-level factors of staff (shared values and understanding, engagement, and communication) [42]. The present study has described a number of micro-level factors within the participating sites, highlighting that the implementation of new models of care should be done at the individual facility level, and not at the health district level, as differences in service delivery between facilities in the same health district do exist.

### 4.3. The ‘So What?’ Question

The present study is somewhat unique amongst the evidence base for discharge planning of older patients, in its use of ethnography to explore ‘on the ground’ processes in real clinical settings. Previous studies have explored the effect of interventions such as comprehensive geriatric assessment on discharge destination or mortality [43], the ability of tools or screening assessments to predict patient outcomes [44], or the experiences of patients and their carers [45]. Identifying models of care that are likely to achieve successful discharge planning of older patients is important, as the need for such services is only going to increase. The number of patients will increase with the aging population, but so will the burden of morbidity in older patients—time to death and morbidity are more strongly associated with health care expenditure than age alone [46]. Given the clear evidence for comprehensive person-centered geriatric assessment to result in increased odds of older patients being alive and in their own homes following hospital admission [43], research now needs to shift to ensuring successful implementation of these services, and on components of discharge planning specifically within that comprehensive assessment. This study may help health policy makers and service managers identify similar issues in discharge planning in their own facilities, provide them with ideas on how to address those issues, and encourage them to consider an evidence-based implementation science approach to reshape their models of care.

## 5. Conclusions

Both hospitals implemented various best-practice interventions to enhance communication, collaboration, and coordination for optimal discharge planning of older patients. Both teams used DCC to facilitate e and encourage appropriate discharge planning for older patients. Clear responsibilities and goals within the MDT helped to create cohesive, well-functioning teams. The active participation of patients and their family in the discharge planning process remains a challenge. Fostering a culture of inclusivity and engagement with patients and families may assist in getting more health professionals to talk to each other about incorporating patient and family perspectives into their clinical practice.

## Data Availability

Not applicable.

## Supplementary Materials

The following supporting information can be downloaded at: https://www.mdpi.com/article/10.3390/healthcare10020202/s1, Supplementary Material S1: Pre-determined codes used for analysis derived from Carroll and Dowling’s essential elements for discharge planning.
