# Supplementary material for: Discharge Planning of Older Persons from Hospital: Comparison of Observed Practice to Recommended Best Practice
Source: Healthcare (Basel). 2022 Jan 20;10(2):202. doi: 10.3390/healthcare10020202 (PMC8872455; doi:10.3390/healthcare10020202)
Supplement: Supplementary file 1 [file healthcare-10-00202-s001.zip › healthcare-1532720-supplementary.pdf]

Supplementary Material S1

Pre-determined codes used for analysis derived from Carroll and Dowling's essential elements for discharge planning.

**Number One: Communication – Clinicians to patient, family/carer, and community service**<br>• Communication should occur between clinicians and the patient, family/carer, and community services (Carroll & Dowling, 2007)<br>• Patient participation, questions from patients/families should be encouraged<br>• Clinicians should communicate consistent messages<br>• Primary care physicians should be involved in the discharge planning process (Kripalani et al., 2007)
**Number Two: Collaboration – Clinicians**<br>• Communication between clinicians is a cornerstone of effective discharge planning (Bull & Roberts, 2001; Carroll & Dowling, 2007)<br>• Communication tools are not enough to ensure effective discharge planning (Olsen & Wagner, 2000)<br>• Each institution should have its own framework to support discharge planning policies and procedures (Carroll & Dowling, 2007)<br>• Characteristics of well-executed multidisciplinary communication: education, negotiation, and exchange of information between health professionals (Allen, Ottmann, & Roberts, 2013)<br>• Characteristics of poorly executed multidisciplinary communication: an absence of understanding of other health professionals' roles, and inadequate dialogue (Allen et al., 2013)
**Number Three: Coordination – by clinician of discharge process; Education – by clinician of patient, family/carer, other clinicians on discharge process**<br>• Coordination and education are essential to achieving successful discharge planning (Carroll and Dowling 2007)<br>• Providing sufficient information through education is a key strategy for promoting successful discharge of frail elderly patients from acute hospitals (Bauer, Fitzgerald et al. 2009)<br>• Designated health personnel or bedside nurses can be employed to coordinate discharge of patients, depending on the preference of the local facility (Guttman, Afilalo et al. 2004, Dedhia, Kravet et al. 2009) (Carroll and Dowling 2007)<br>• Other facilities employ screening tools to identify patients with complex discharge needs, checklists to remind staff of recommended procedures, and information booklets for patients and families (Carroll and Dowling 2007)
**Number 4: Patient participation – goal setting**<br>• Involving patients in the planning of their own discharge is necessary for the transition from hospital to home to successfully occur (Carroll & Dowling, 2007)<br>• Patients should be empowered and actively involved in the discharge planning process (Carroll & Dowling, 2007)<br>• Patients should not be talked about, reduced to a passive position, or isolated through medical jargon (Carroll & Dowling, 2007), (Bangsbo, Duner, & Liden, 2014)<br>• Patients should be made ready for discharge with interventions that target education and patient decision-making (Mabire, Bula, Morin, & Goulet, 2015)
